# It is unlikely that oxygen supplementation in COPD patients with chronic respiratory failure reduce cardiac troponin level

**DOI:** 10.1186/s12890-022-02169-7

**Published:** 2022-11-01

**Authors:** Natalia Kononova, Gunnar Einvik, Nils Henrik Holmedahl, Tor-Arne Hagve, Torbjørn Omland, Vidar Søyseth

**Affiliations:** 1 Faculty of Medicine, Institute of Clinical Medicine, University of Oslo, Oslo, Norway; 2 Department of Pulmonology, Division of Medicine, Akershus University Hospital, 1470 Lørenskog, Norway; 3 LHL Hospital Gardermoen, Oslo, Norway; 4 Unit of Medical Biochemistry, Division of Diagnostics and Technology, Akershus University Hospital, Lørenskog, Norway

## Abstract

**Background:**

Cardiac troponin T (cTnT) is a biomarker of myocardial injury frequently elevated in COPD patients, potentially because of hypoxemia. This non-randomised observational study investigates whether long-term oxygen treatment (LTOT) reduces the cTnT level.

**Methods:**

We compared cTnT between COPD patients who were candidates for LTOT (n = 20) with two reference groups. Patients from both reference groups were matched with the index group using propensity score.Reference groups consists of institutional pulmonary rehabilitation patients (short-term group) (n = 105 after matching n = 11) and outpatients at a pulmonary rehabilitation clinic (long-term group)(n = 62 after matching n = 10). Comparison was done within 24 h after LTOT initiation in first reference group and within 6 months after inclusion in the second group.

**Results:**

The geometric mean of (standard deviation in parentheses) cTnT decreased from 17.8 (2.3) ng/L (between 8 and 9 a.m.) to 15.4 (2.5) ng/L between 1 and 2 p.m. in the LTOT group, and from 18.4 (4.8) ng/L to15.4 (2.5) ng/L in group (1) The corresponding long-term results were 17.0 (2.9) ng/L at inclusion (between 10 and 12 a.m.) to 18.4 (2.4) ng/L after 3 months in the LTOT-group, and from 14.0 (2.4) ng/L to 15.4 (2.5) ng/L after 6 months in group (2) None of the differences in cTnT during the follow-up between the LTOT-group and their matched references were significant.

**Conclusion:**

Initiation of LTOT was not associated with an early or sustained reduction in cTnT after treatment with oxygen supplementation.

**Supplementary Information:**

The online version contains supplementary material available at 10.1186/s12890-022-02169-7.

## Background

Cardiac troponins T (cTnT) and I (cTnI) in peripheral circulation are highly specific and sensitive biomarkers of myocardial cell damage. The troponin complex is composed of three proteins: troponin T, troponin I, troponin C (TnT, TnI and TnC ). It is located on the thin filament of the contractile apparatus in all types of striated (skeletal and cardiac) muscle, but is not found in the smooth muscle [1].

Chronic obstructive pulmonary disease (COPD) is a progressive disease characterised by irreversible airflow limitation and associated with an enhanced inflammatory response to noxious particles or gases in the airways and the lung [2].One important and dangerous complication of COPD is chronic respiratory failure. This is a syndrome in which the respiratory system fails in one or both of its gas exchange functions: oxygenation and carbon dioxide elimination of the blood. Prognosis is quite poor and Long-Тerm Оxygen Тreatment (LTOT) is a recommended treatment to improve survival and quality of life of hypoxemic COPD patients [2].

Previous retrospective and prospective studies have shown elevations of cardiac troponin during COPD exacerbations[3, 4] and in a stable disease [5]. cTnT in COPD patients is associated with increased mortality, independently of presence of cardiac disease[3, 5–7].

However, the underlying pathophysiological mechanisms of cardiac troponin (cTn) release in COPD are still incompletely understood. Possible mechanisms include physiological stressors such as tachycardia and hypoxemia leading to subclinical ischemic injury and leakage of cTn to the circulation.

We hypothesized that treatment of hypoxemia by supplementary oxygen may oppose and reduce ischemic injury, and thereby reduce cTn in the circulation.

Therefore, in the present study we aimed to test this hypothesis by assessing the short-term and long-term effects initiating of LTOT on circulatory cTnT concentration in stable COPD patients with chronic respiratory failure.

## Materials and methods

### Study population and design

This is a “non-randomised study” of the effect of oxygen supplementation on cTnT in COPD patients with respiratory failure. Inclusion criteria were diagnosed COPD (forced expiratory volume in one second (FEV_1_) / forced vital capacity (FVC) < 70% after inhalation of 400 µg salbutamol), age above 40 years and a cumulative tobacco consumption of 10 pack-years or more. For the intervention group, the following clinical criteria for initiation of LTOT were used:

1. Resting partial pressure of arterial oxygen [PaO_2_] of < 7.3 kPa (55 mmHg).
2. PaO2 < 8.0 kPa (60 mm Hg) in case of concomitant signs of right-sided heart failure, pulmonary hypertension or secondary polycythemia.
3. Non-smokers for at least three months, adjudicated by the attending physician.

Exclusion criteria for the study were: history of asthma or other lung diseases, current exacerbation of COPD and absence of competence to provide writing consent to participation.

**Fig. 1:**
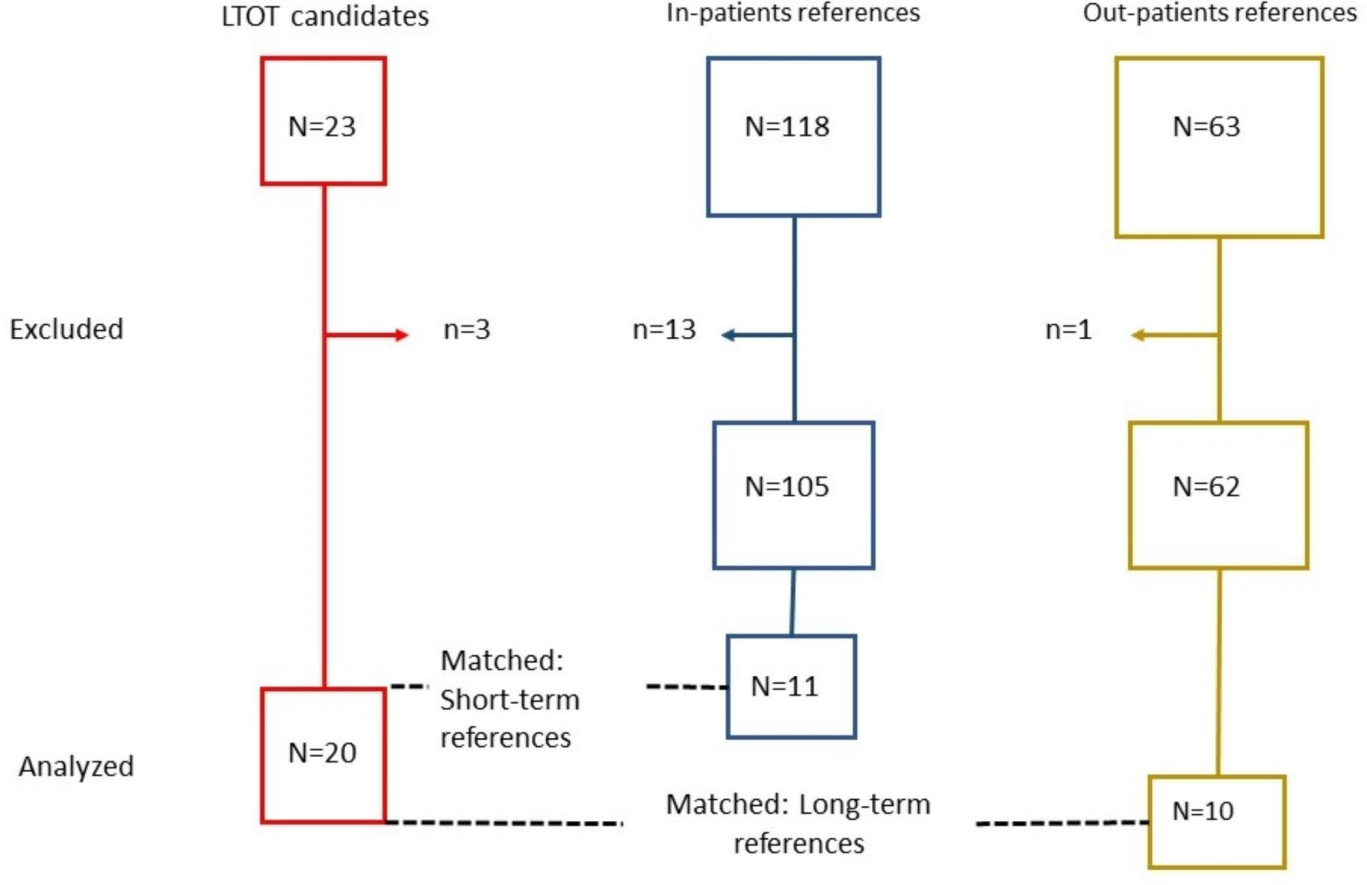
Flowchart showing the long-term oxygen treatment group (n = 20), the short-term (n = 11), and the long-term references, respectively, were selected.

A consecutive set of 23 COPD patients electively admitted to the Pulmonary Department, Akershus University Hospital), Norway, were considered for eligibility for the intervention group. A total of 3 patients were not accepted for LTOT: one patient developed CO_2_-retension, one patient had used tobacco < 3 months and one suffered from a current COPD exacerbation. The remaining 20 patients were included in the intervention group in the present study (Fig. 1). Among these 18 patients followed-up at three months after inclusion.

The LTOT candidates followed the clinical routine with a 24 h hospitalisation during initiation of LTOT to test their tolerance for oxygen. They were readmitted after 3 months for reassessment of oxygen dose. An ambulatory team (pulmonary nurse and physiotherapist) visited the patients between these two hospitalizations to facilitate the use of LTOT.

The observational references were enrolled from an institutional COPD rehabilitation program at LHL-hospital Glittre (In-patients references) (n = 118), and from an outpatient COPD rehabilitation program at Akershus University Hospital (out-patients references) (n = 63). Thirteen patients were excluded from the in-patients reference group: 10 patients were LTOT-users, and six (three of them were LTOT-users) patients did not have measurement of cTnT at all the three time points (see data collection). The out-patients references were reassessed 6 months after inclusion as a part of their rehabilitation program. In this group one patient was excluded due to LTOT (Fig. 1).

The flowchart shows the patients who started LTOT and gave their consent for participation in the study (n = 20), their matched short-term references (n = 11), and the matched long-term references (n = 10).

Flowchart showing the long-term oxygen treatment group (n = 20), the short-term (n = 11), and the long-term references (n = 10), respectively, were selected.

The characteristics of source cohorts of the study are shown in Table 1, and the final LTOT group with their matched controls are shown in Table 2.

**Table 1:** Baseline demographic and clinical data in COPD patients who were candidates for long-term oxygen treatment (LTOT), among COPD patients who participated in outpatient clinic rehabilitation and an institutional rehabilitation program for COPD patients

Covariates	LTOT (n = 23)	Outpatients (n = 63)	Institutional (n = 118)	p-value
Demographic data				
Female, n (%)	16 (70)	28 (46)	67 (54)	0.165
Age, years, mean (sd)	71.9 (7.1)	65.8 (7.2)	64.1 (7.9)	< 0.001
BMI, kg/m², mean (sd)	25.2 (7.2)	25.4 (5.4)	24.7 (5.6)	0.693
Coronary Heart Disease, n (%)	2 (10)	11 (18)	12 (10)	0.300
Smoking history				
Pack years, mean (sd)	46.0 (31.6)	42.3 (20.9)	35.0 (18.5)	0.020
Current, n (%)	0 (0)	27 (44)	23 (19)	< 0.001
Clinical data				
SBP, mmHg, mean (sd)	129 (17.9)	132 (28.0)	138 (18.4)	0.089
Arterial HT, n (%)	14 (61)	29 (48)	49 (42)	0.234
LTOT-users, n (%)	0 (0)	1 (2)	13 (11)	0.015
Diabetes mellitus, n (%)	1 (4)	10 (16)	7 (6)	0.074
Pulmonary function, mean (sd)				
Spirometry				
FVC, Litres	2.1 (0.79)	3.1 (0.96)	2.5 (0.87)	< 0.001
FEV1, Litres	1.0 (0.70)	1.5 (0.58)	1.1 (0.52)	< 0.001
FEV1/FVC, %	50 (24)	53 (14)	44 (10)	0.001
Arterial blood gases				
Oxygen-tension, kPa	7.6 (1.1)	10.6 (2.3)	8.9 (1.4)	< 0.001
CO2-tension, kPa	5.6 (0.90)	4.9 (0.78)	5.7 (0.91)	< 0.001
CAT-score	22.0 (10.6)	19.0 (7.8)	18.4 (6.2)	0.109
Laboratory data, gm (gsd)				
Haemoglobin, g/dL	14.3 (2.0)	14.3 (1.6)	14.5 (1.3)	0.575
Leucocytes, 10^9^/L	7.3 (1.5)	6.8 (1.7)	7.6 (1.3)	0.167
C-Reactive Protein, mg/L	5.1 (4.1)	3.2 (2.5)	2.6 (3.0)	0.021
Creatinine, µmol/L	68.2 (1.5)	74.8 (1.2)	75.0 (1.2)	0.241
cTnT, ng/L	16.6 (2.8)	7.1 (2.2)	3.9 (3.4)	< 0.001
Electrocardiogram, n (%)				
Pathological Q-wave	3 (13)	8 (13)	11 (9)	0.738
Left Bundle Branch Block	1 (4)	1(2)	2 (2)	0.721

BMI; Body mass index, SBP: Systolic Blood pressure, HT: Arterial Hypertension, FVC: Forced vital capacity, FEV1: Forced expiratory volume in one second, CAT: COPD assessment test, cTnT: Cardiac troponin T, sd: Standard deviation, gm: geometric mean, gsd: geometric standard deviation

**Table 2:** Distribution of baseline covariates in the long-term oxygen treatment group, the matched short-term, and the matched long-term references with the corresponding standardised differences (St-D) between the LTOT-group and the references. Note that some of the references are counted several times so that number of observation is 20 in each group (index to references = n to 1)

	Treatment	Reference groups			
**Covariates at inclusion**	**LTOT**	**Short-term**	**St-D**	**Long-term**	**St-D**
Female, N (%)	14 (70)	15 (75)	0.11	13 (65)	0.15
Age, years, mean (sd)	72.5 (6.8)	70.9 (6.9)	0.08	71.5 (3.2)	0.06
Arterial hypertension, n (%)	10 (60)	11 (65)	0.10	7 (44)	0.33
Diabetes, n (%)	1 (5)	0 (0)	0.32	3 (19)	0.44
FEV1, Litres, mean (sd)	0.93 (0.65)	0.79 (0.37)	0.09	1.06 (0.44)	0.08
ISWT, m, mean (sd)	109 (101)	n.a.		116 (89)	0.03
CO2-tension, kPa, mean (sd)	5.7 (0.88)	5.9 (1.1)	0.08	5.4 (1.1)	0.11
CAT-score, mean (sd)	23.7 (901)	24.7 (4.1)	0.05	21.4 (4.6)	0.11
Leucocytes, 10^9^/Litres, gm (gsd)	7.8 (2.9)	9.2 (4.2)	0.15	6.5 (1.0)	0.20
C-Reactive Protein, mg/L, gm (gsd)	5.1 (4.4)	5.9 (4.0)	0.04	2.7 (3.5)	0.17
Creatinine, µmol/Litres, gm (gsd)	69.4 (1.5)	75.3 (1.3)	0.08	61.6 (1.2)	0.13
hs-cTnT, ng/L, gm (gsd)	16.5 (2.7)	20.7 (4.9)	0.06	14.0 (2.4)	0.06

sd: standard deviation, FEV1: Forced Expiratory Volume in one second, ISWT: Incremental Shuttle Walk Test, n.a.: not available. CAT: COPD Assessment Test, gm: geometric mean, gsd: geometric standard deviation, CRP: C-Reactive protein, hs-cTnT: high-sensitivity cardiac troponin T

### Data collection

At inclusion, all participants completed a self-administered respiratory questionnaire including respiratory symptoms, smoking habits, medical history, and medical treatment. In addition, the patients underwent a clinical examination including COPD assessment test (CAT) [9], examination of lung function, walking distance, electrocardiography, venous blood sampling and arterial blood gases at inclusion as well as at the end of the follow-up. Blood samples were collected at three time points:

- T1: between 8 and 9 a.m. – the intervention group and institutional references.
- T2: between 10 and 12 a.m. – all three groups.
- T3: between 1 and 2 p.m. – the intervention group and institutional references,

*Spirometry and reversibility test*: Spirometry was performed as recommended by the European Respiratory Society [10] at the respiratory laboratories at Akershus University Hospital and Glittre Hospital. The patients underwent a reversibility test using 400 µg salbutamol. Spirometry was repeated after 15 min to provide post bronchodilator (BD) FEV_1_/FVC-ratio to assess the eligibility. *Exercise test*: In the intervention group and the outpatients, physical exercise capacity was investigated using the incremental shuttle walk test (ISWT) as described by Singh et al. [11]. In the in-hospital rehabilitation group, exercise test was performed using 6 min walking test.

#### Arterial blood gases

Arterial tension of oxygen (pO2) and carbon dioxide (pCO2) were measured on Radiometer ABL720Flex (Radiometer, Copenhagen, Denmark).

#### Blood sampling and biochemical assays

Venous blood samples were collected and centrifuged and *s*erum and plasma were aspirated within 60 min, and immediately stored at -80^o^C for subsequent analyses of cTnT with a high-sensitivity assay (Cobas e 8000 immunoanalyser, Roche Diagnostics, Mannheim, Germany). The lower limit of detection for this assay was 3.0 ng/l. C-reactive protein (Cobas c501/502 system, Roche, Mannheim, Germany) was analysed consecutively and had a lower limit of detection of 3.0 mg/L. Haemoglobin, leukocyte count and creatinine were also analysed consecutively by the hospitals’ routine methods.

### Endpoints

The primary endpoint was the change in circulating concentrations cTnT from inclusion to the end of the follow-up (long-term effect by LTOT). The secondary endpoint was the serial change in venous cTnT between T1 and T3 (short-term effect of LTOT).

### Statistics

Demographic data at baseline were analysed using Chi-square, two-sample t-test, and one-way Analysis of Variance as appropriate. Due to a skewed distribution, the main outcome, cTnT, was log-transformed.

The data were analysed using propensity score with one-to-one matching with replacement [12]. Briefly, propensity score has been described and defined by Rosenbaum and Rubin to be the probability of treatment assignment conditional on observed baseline covariates [13]. We estimated this probability using logistic regression with LTOT at inclusion as the outcome variable. We developed one model for the short term-effect, and one model for the long-term effect. In both models we included baseline covariates that were associated with the LTOT assignment, i.e. FEV1, COPD assessment test (CAT-score), arterial carbon dioxide tension, shuttle walk distance (only the LTOT-group and the out-patient clinic patients) or associated with cTnT-level at inclusion, i.e. arterial hypertension, history of coronary arterial disease, diabetes, creatinine, and cTnT at inclusion, in the initial model. In addition, we included gender, age, total leucocyte count, and C-reactive protein (CRP) as covariates.

Smoking habits and oxygen tension were not included in the initial models, as they were criteria for LTOT.

The model was then reduced by backward elimination of non-significant covariates provided that the fitted data did not decrease the area under the ROC curve, and the p-value of the Hosmer-Lemeshow test was non-significant. The propensity score, i.e. the probability of LTOT, was estimated as the predicted logit from the final model. We developed two propensity score models (ps-models); both with LTOT as the outcome, using covariates from the LTOT-group and the in-patients reference group (ps-model 1), and covariates from the LTOT and the out-patients reference group (ps-model 2). *Thereby, two sets of propensity scores were established.*

In each model we used one-to-one matching (pair matching) with replacement, i.e. allowing for many-to-one matches between the referents and the index cases. The lowest difference in propensity score was used as the matching criterion. The similarity of the LTOT group and the matched references was assessed using standardized differences [14]. A standardized difference less than 0.1 indicate negligible differences between the groups [15].

We used the change in cTnT between T1 and T3 as the outcome in the first model (diurnal effect), and the change in cTnT from baseline to follow-up at 3 and 6 months, respectively, in the second model. As there were several cases for each reference, the differences in cTnT-changes between the groups were tested using robust estimates of standard errors. In all these analyses with cTnT as the dependent variable, cTnT we used in the log-transformed form. Furthermore, we used ordinary least square regression with robust standard errors.

### Sample size

The sample size was based on our previous study [7]. We considered 25% decrease in cTnT be considered as a clinical meaningful effect of LTOT. We regarded 25% decline in cTnT as a clinical meaningful reduction after commencement of oxygen supplementation. Based on this assumption, we calculated that we needed to include 20 patients in order to detect a reduction in cTnT of 25% with significance level = 0.05 and power = 0.90. Reference group 1 and 2 were established to adjust for the natural diurnal variation and the long-term change in cTnT, respectively.

All statistical analyses were performed using Stata/SE 15.1, StataCorp LCC, Texas, USA.

## Results

### Patient characteristics

In the source populations, the LTOT candidates were older and had a more severe smoking history. They had lower lung function, lower arterial oxygen tension, higher carbon dioxide tension, higher CRP and considerably higher cTnT concentrations compare to the reference groups (Table 1).

After matching with replacement it turned out that 10 references in the long-term group and 11 references in the short-term references minimalized the difference in propensity score between the LTOT-group and the respective references. Thereby n = 11 from the short-term references, and n = 10 from the long-term references were used in the final analyses. However, compared with the matched references the differences between LTOT-group and the matched short-term as well as the long-term reference, respectively, were negligible (Table 2).

Moreover, the propensity score models fitted the data well (supplemental Table 1), especially regarding the most significant determinants of LTOT (Table 2). Foremost, the cTnT-level showed an improved similarity between the LTOT-group and both references.

### Short-term effects

There was a significant short-term decrease in cTnT from T1 to T2 in both groups combined, as well as in the LTOT group separately, but not in the short-term references. However, the decline in cTnT from 8 to 9 a.m. to 1–2 p.m. did not differ between the intervention group and short-term references (Table 3).

**Table 3:** Geometric means of high-sensitivity of cardiac troponin T (hs-cTnT) in patients that started long-term oxygen supplementation (LTOT: yes), compared to matched short-term and long-term reference groups (LTOT: no)

Time frame	Both groups: ng/L gm (gsd)	LTOT: yes, ng/L gm (gsd)	LTOT: no, ng/L gm (gsd)	LTOT: yes/no^a^ Ratio (SE), p-value
Long-term	N = 26	n = 18	n = 8	
I: Inclusion	15.5 (2.6)	16.5 (2.7)	14.0 (2.4)	
F: Follow-up	16.8 (2.3)	18.4 (2.4)	15.2 (2.3)	
Ratio F/I, p-value	1.08, 0.126	1.08, 0.346	1.09, 0.226	
				0.99 (1.11), 0.903
Short-term	N = 32	n = 20	n = 12	
T1: 08–09 a.m.	18.1 (3.5)	17.8 (2.3)	18.4 (4.8)	
T3: 13–15 p.m.	15.9 (3.8)	15.4 (2.5)	16.4 (5.4)	
Ratio T3/T1, p-value	0.88, < 0.001	0.87, 0.005	0.89, 0.103	
				0.97 (1.08), 0.741

^a^The ratio between the baseline (cTnT_0_) to follow-up (cTnT_1_) change in hs-cTnT in the Index group to that of the reference groups, given by: ((cTnT_1_)/( cTnT_o_))_Index group_/((cTnT_1_)/( cTnT_o_))_References_ Gm: Geometric mean, gsd: geometric standard deviation, SE: standard error

### Long-term effects

There was a numerical increase on cTnT-level in both groups from inclusion to the end of the long-term follow-up. Moreover, the change in cTnT during the follow-up did not differ between the groups (Table 3).

For evaluation of LTOT compliance we assessed the PaO2 levels at T4. PaO2 increased from 7.6 (SD 1.1) kPa to 9.2 (1.7) kPa in the LTOT group (p = 0.002), but was stable in the matched reference group: 10.4 (SD 1.8) kPa at inclusion to 10.3 (SD 1.5) kPa (p = 0.931) at the follow-up.

## Discussion

The main finding in our study is that reversing hypoxemia in chronic respiratory failure did not find an association between LTOT and troponin changes on short and long term .

To the best of our knowledge, this is the first study prospectively measuring cTnT in stable COPD patients with chronic respiratory failure before and after LTOT treatment. LTOT-candidates are characterised by severe arterial hypoxemia. It has been suggested that arterial hypoxemia may explain the troponin elevation in these patients, as well as COPD patients in general [16]. Moreover, it is well accepted that elevated troponin is a predictor of mortality in COPD, chronic coronary heart disease, and in general populations [17–19]. We hypothesized that hypoxemia would be the main etiological factor for myocardial damage and thus cTn levels in COPD. Therefore, we expected that LTOT should reduce the cTn level. However, in our study cTnT did not decrease within three months after commencement of LTOT. On the contrary, cTnT slightly increased (numerical increase) during these months. Several explanations should be considered: First, low compliance could be a possible explanation. However, all the patients in our study underwent training in using LTOT during their stay in hospital and a follow-up at home. Moreover, they were regularly visited and contacted by phone by an Ambulatory Team consisting of nurses and physical therapist in regards to use of LTOT. Blood tests for cTnT measurements were always collected at the hospital where use of oxygen was carefully checked. Also, a significant increase in PaO2 was recorded in LTOT patients at the follow-up after 3 months [20]. Finally, our hypothesis that oxygen supplementation is associated with a decrease in cTnT may be incorrect. Secondly, the lack of effect of LTOT on cTnT could potentially be explained by irreversible myocardial injury caused by prolonged hypoxemia leading to permanent elevation of troponin level that is unresponsive to oxygen supplementation.

The choice of propensity score as the analytical approach merits some comments. First and foremost, the cTnT-levels as other indices of COPD severity differed greatly between the groups. After matching references to the treatment group, these dissimilarities attenuated markedly. This matching process is driven by the matching rules, whereas the regression adjustment maybe guided by the results such as selection of covariates. However, during the matching process, the majority of the information in the data are not used but it can be claimed that the references that are not used do not contribute with any relevant information. Moreover, matching with replacement result in multiple use of some references thereby increasing the type-I error. This problem was solved using robust standard errors. Matchings with replacement, instead of without replacement is that when the matches are done without replacement the sorting order of the observations would matter to find the matches. This is unfortunate, especially when there are ties in the matches and hence we have no theoretical ground which matches to choose among the ties for the current observation.

Two other observations in this study also merit comments. First, the cTnT concentrations varied through the day with significantly lower concentrations in both groups at midday compared to the morning. Such diurnal variations have been observed in other cohorts as well (Klinkenberg et al., 2016), and may indicate that this is a general phenomenon, characterized by gradually declining concentrations during daytime followed by rising concentrations at night peaking the early morning hours.

Secondly, during follow up on a long-term basis cTnT increased in both groups. This observation can indicate that the patients’ cTnT level followed the natural course independently of O2-supplementation. Another hypothesis to explain increases of cTnT may be increased systemic inflammation associated with accelerated airflow limitation [21–23], which in the present study was supported by a correlation between CRP levels and FEV1. It has also been suggested that COPD and CVD involve an acceleration of the normal ageing process which links to low-grade chronic systemic inflammation [24]. Elevated cTnT may be an expression for this process independent of hypoxemia.

The main limitation of the study could be the small number of patients treated by LTOT, thereby limiting the external validity of the results. Small samples are liable to sampling bias distorting the results. However, there was a large variability of cTnT in the LTOT group indicating that that the sample was unbiased. Next, the intervention group had elevated cTnT at inclusion. Therefore, a regression to the mean effect should likely reduce cTnT to normal levels but we did not observe such an effect. Moreover, we believe that inclusion of reference groups adjusts for the natural course of troponin during the day and the follow-up.

The study design was not optimal. The follow-up time was different in the LTOT group (3 months) and long-term reference group (6 months). The times were chosen because of practical reasons related to hospital routine control but we do not believe that this difference explains our results. Since the long-term references had a longer observation time than the LTOT group, the potential time bias should be taken into account.

Ideally, the effect of oxygen treatment on cTn should be investigated using a randomised clinical trial (RCT) design. However, LTOT is an established life-saving treatment in COPD with respiratory failure that preclude RCT to investigate if LTOT reduces cTn. Consequently, we believe that some kind of observational design must be chosen.

## Conclusion

It is unlikely that LTOT reduce the cTn level in the stable COPD patients with chronic respiratory failure.

## Electronic supplementary material

Below is the link to the electronic supplementary material.

Supplementary Material 1

## Data Availability

The datasets that were used and analysed during the current study are available from the corresponding author on reasonable request. The data that support the findings of this study are available from the corresponding author, Vidar Søyseth, upon reasonable request.
