# Supplementary material for: It is unlikely that oxygen supplementation in COPD patients with chronic respiratory failure reduce cardiac troponin level
Source: BMC Pulm Med. 2022 Nov 1;22:392. doi: 10.1186/s12890-022-02169-7 (PMC9623981; doi:10.1186/s12890-022-02169-7)
Supplement: Supplementary file 1 — Supplementary Material 1 [file 12890_2022_2169_MOESM1_ESM.docx]

Supplemental table 1. Logits for commencing long-term oxygen treatment (β), based on relevant covariates in the short-term and long-term models by relevant covariates, and post estimation test results.

|  | Short-term model | | | Long-term model | | |
| --- | --- | --- | --- | --- | --- | --- |
| Covariates | Β | se | p-value | β | se | p-value |
| Intercept | -11.0 | 3.3 | 0.002 | -11.0 | 5.4 | 0.036 |
| Age, years | 0.14 | 0.051 | 0.008 | 0.077 | 0.065 | 0.194 |
| Gender: F vs. M | 1.99 | 0.84 | 0.017 | 2.56 | 1.12 | 0.022 |
| Carbon dioxide-tension, kPa | -0.59 | 0.42 | 0.156 | 0.77 | 0.39 | 0.048 |
| Log(CRP, mg/L) | n.i. |  |  | 0.054 | 0.031 | 0.079 |
| Log(hs-cTnT, ng/L) | 1.07 | 0.28 | < 0.001 | 0.77 | 0.53 | 0.141 |
| ISWT, in quartiles | n.a. |  |  | -1.64 | 0.62 | 0.008 |
|  |  |  |  |  |  |  |
| Post-estimation |  |  |  |  |  |  |
| Hosmer-Lemeshow, p-value | 0.990 |  |  | 0.999 |  |  |
| Sensitivity, % | 55 |  |  | 80 |  |  |
| Specificity, % | 93 |  |  | 91 |  |  |
| Correctly classified, % | 86 |  |  | 89 |  |  |
| AUC-ROC, (standard error) | 0.91 (0.030) | |  | 0.95 (0.022) | |  |

se: standard error, CRP: C-Reactive protein, hs-cTnT: high-sensitivity cardiac troponin T, ISWT: Incremental shuttle walk test, n.i.: not included, n.a.: not available, AUC-ROC: Area under the receiver operating curve.
